# Clinical usefulness of T-SPOT.TB in the diagnosis tuberculosis in emergency patients

**DOI:** 10.1186/1471-227X-12-S1-A7

**Published:** 2012-12-18

**Authors:** HU Xiao-min, LIU Jian, LEI Long, YIN Wen

**Affiliations:** 1 Department of Emergency Xijing Hospital, the Fourth Military Medical University, Changle Xilu Road No.127, Xi'an 710032, China

## Objective

To evaluate the diagnostic value of an interferon-gamma release assay T-SPOT.TB in tuberculosis of emergency patients.

## Methods

The emergency patients with suspected tuberculosis received T-SPOT.TB assay in the peripheral blood mononuclear cells(PBMCs).

## Results

T-SPOT.TB assay, PPD, anti-TB antibody test, M tuberculosis polymerase chain reaction, acid-fast bacilli stain and cultures for M tuberculosis were positive in 96.8%(31/32), 43.7%(14/32), 21.8%(7/32), 15.6%(5/32), 13.0%(3/23) and 4.3%(1/23), respectively, of patients with active tuberculosis. The sensitivity and specificity of the T-SPOT.TB were 96.8% and 98.3%.

## Conclusions

T-SPOT.TB assay is a rapid and high-sensitive tests for diagnosing tuberculosis in emergency patients.

